# Efficacy of bevacizumab through an indwelling pleural catheter in non-small cell lung cancer patients with symptomatic malignant pleural effusion

**DOI:** 10.1186/s12890-024-02886-1

**Published:** 2024-02-16

**Authors:** Hao Zeng, Yuanyuan Zhang, Sihan Tan, Qin Huang, Xin Pu, Panwen Tian, Yalun Li

**Affiliations:** 1 Lung Cancer Center/Lung Cancer Institute, West China Hospital , Sichuan University, No. 37 GuoXue Alley, 610041 Chengdu, Sichuan Province China; 2 Department of Pulmonary and Critical Care Medicine, State Key Laboratory of Respiratory Health and Multimorbidity, Precision Medicine Key Laboratory of Sichuan Province, West China Hospital, Sichuan University, 610041 Chengdu, Sichuan China

**Keywords:** Bevacizumab, Indwelling pleural catheter, Non-small cell lung cancer, Malignant pleural effusion, Actionable mutations

## Abstract

**Background:**

Several studies have indicated that intrapleural infusion of bevacizumab is an effective treatment for non-small cell lung cancer (NSCLC) with malignant pleural effusion (MPE). However, the impact of bevacizumab administered through an indwelling pleural catheter (IPC) on the prognosis of these patients is unknown.

**Methods:**

Consecutive advanced NSCLC patients with symptomatic MPE receiving an IPC alone or bevacizumab through an IPC were identified in a tertiary hospital. The patient characteristics and clinical outcomes were collected.

**Results:**

A total of 149 patients were included, and the median age was 60.3 years. Males and nonsmokers accounted for 48.3% and 65.8%, respectively. A total of 69.8% (104/149) of patients harbored actionable mutations, including 92 *EGFR*-activating mutations, 11 *ALK* fusions, and 1 *ROS1* fusion. A total of 81.9% (122/149) of patients received IPC alone, and 18.1% (27/149) received bevacizumab through an IPC. The incidence of spontaneous pleurodesis during the first 6 months was greater in the bevacizumab-treated group than in the IPC-treated group in the subgroup with actionable mutations (64.3% vs. 46.9%, *P* = 0.28). The median overall survival (OS) in patients with actionable mutations treated with bevacizumab through an IPC was 42.2 months, which was significantly longer than the 26.7 months in patients who received an IPC alone (*P* = 0.045). However, the median OS did not differ between the two arms in the subgroup without actionable mutations (10.8 vs. 41.0 months, *P* = 0.24). No significant difference between the bevacizumab through an IPC group and the IPC group was detected in the number of participants who had adverse events, either in patients with actionable mutations (14.3% vs. 8.4%; *P* = 0.42) or in patients without actionable mutations (16.7% vs. 12.8%; *P* = 1.00).

**Conclusions:**

Bevacizumab through an IPC resulted in a significantly improved prognosis for NSCLC patients with MPE and actionable mutations. However, patients without actionable mutations do not benefit from bevacizumab through IPC.

**Supplementary Information:**

The online version contains supplementary material available at 10.1186/s12890-024-02886-1.

## Introduction

Malignant pleural effusion (MPE) is a common complication of advanced tumors [1] that accounts for approximately 126,800 hospital admissions per year in the USA and is the second most common cause of pleural effusion in China [2, 3]. Lung cancer is the leading cause of MPE, and approximately one-third of MPE cases are caused by non-small cell lung cancer (NSCLC) [4]. MPE was associated with increased morbidity and mortality. Generally, the average survival of patients with MPE is only 3–12 months [5, 6].

Currently, the standard treatment strategy recommended for MPE is still the use of an indwelling pleural catheter (IPC), especially in China, where talc is unavailable [7, 8]. Since the occurrence of MPE indicates advanced disease, the standard treatment strategies are mostly palliative and do not improve the prognosis of NSCLC patients with MPE [7, 9].

With advances in the understanding of the pathogenesis of MPE [10, 11], intrapleural administration of bevacizumab has become a new treatment strategy for MPE [5, 12]. Several clinical trials have shown that intrathoracic injection of bevacizumab is effective at controlling NSCLC-induced MPE [10, 12, 13]. Du N et al. [10] compared the efficacy of intrapleural administration of bevacizumab plus cisplatin with that of cisplatin alone for NSCLC-mediated MPE and reported a significantly greater response rate in the combination treatment group. A randomized trial also reported a higher MPE remission rate in patients who were intracavitary perfused with bevacizumab and cisplatin than in those with cisplatin alone [14]. Although intracavitary infusion of bevacizumab may improve the MPE response rate, there is no evidence to show whether bevacizumab administered through an IPC can prolong overall survival (OS). Additionally, large, randomized studies recently reported that the addition of bevacizumab to EGFR tyrosine kinase inhibitors (TKIs) substantially improved PFS in patients with *EGFR*-mutant NSCLC [15, 16]. A randomized CTONG1509 phase III study [16] indicated that patients with *EGFR* L858R-related mutations derived more benefits from bevacizumab plus erlotinib than did those with a deletion within exon 19. Therefore, it is also important to understand how specific mutations might affect the efficacy of bevacizumab.

Therefore, we conducted a retrospective study to assess the impact of bevacizumab through an IPC on the prognosis of patients with NSCLC and MPE and sought to evaluate whether the impact was different in patients with actionable mutations than in those without actionable mutations.

## Methods

### Patients and data collection

We included consecutive patients with NSCLC and MPE who received bevacizumab through an IPC or who had an IPC alone between January 2015 and December 2022 at West China Hospital of Sichuan University. Histological diagnosis was evaluated based on the 2015 World Health Organization Classification of Lung Tumors, and the tumor stage was evaluated according to the eighth edition of the TNM staging system [17, 18]. The key eligibility criteria were pathologically confirmed NSCLC, MPE diagnosis by cytology and/or histological examination of pleural biopsy tissue, completion of at least one cycle of bevacizumab treatment through an IPC, and no history of IPC placement or intrapleural administration of bevacizumab. Patients were excluded if they were treated with intrapleural chemotherapy or without treatment. Clinical data at the time of treatment initiation, the time of MPE recurrence requiring intervention, and information on clinical follow-up were collected through medical records. Patients were divided into two subgroups based on whether they had oncogenic driver mutations; the actionable mutation group and the no actionable mutation group were defined as patients with or without sensitizing *EGFR* mutations or *ALK/ROS1* fusions, respectively.

Our study was carried out according to the Declaration of Helsinki (2013 EDITION). This study was approved by the Ethics Committee of West China Hospital (No. 2022 − 1085). The need to obtain individual consent for this study was waived by the Ethics Committee of West China Hospital, as the privacy of the patients was not disclosed.

### Detecting EGFR/ALK/ROS1

Sensitizing *EGFR* mutations or *ALK/ROS1* fusions were detected by DNA-based next-generation sequencing (NGS). The NGS-detected samples included histological specimens and MPE samples.

### Treatment and assessments

All procedures were performed at the bedside with B-ultrasound guidance. Under appropriate local anesthesia, an IPC was inserted into the intercostal space, which was subsequently attached to a thoracic drainage bag. In the patients in the IPC group, MPE was drained via the IPC every day. After the MPE was fully drained through the IPC, patients in the bevacizumab through IPC group were given bevacizumab (100 mg-300 mg) dissolved in 60 ml of 0.9% saline solution through the IPC once every three weeks for one to four cycles. Patient rotation is not necessary after intrapleural instillation of bevacizumab.

All of the patients received systemic anticancer therapy as a first-line treatment according to established guidelines [19, 20]. The radiological response of the tumors was evaluated using computed tomography (CT) and brain magnetic resonance imaging (MRI) every 8–10 weeks. The evaluation of MPE was based on thoracic CT or ultrasound. The cutoff date was January 2023; patients without radiographic disease progression at the latest date were considered censored.

Spontaneous pleurodesis was defined based on previous studies [21, 22] and was defined if there was a lack of ipsilateral reaccumulation of MPE and if the patient did not require intervention for ipsilateral MPE during the follow-up period after IPC removal; pleurodesis failure was defined as recurrent and symptomatic ipsilateral MPE that needed pleural intervention within the follow-up period after IPC removal.

The efficacy assessment was based on the Response Evaluation Criteria in Solid Tumors (RECIST) version 1.1 [23]. As defined previously [24, 25], the disease control rate (DCR) was calculated as the percentage of patients with CR plus PR plus stable disease (SD) among all patients. OS was defined as the period from the date of treatment initiation until death from any cause or the last follow-up.

### Statistical analysis

Continuous variables are presented as medians and interquartile ranges (IQRs). Frequencies and percentages were used to describe categorical variables. Baseline characteristics were compared between patients who received bevacizumab through an IPC or who had an IPC alone using the chi-square test or Fisher’s exact test. Differences in the incidences of spontaneous pleurodesis and DCR were compared by the chi-square test. OS was estimated using the Kaplan‒Meier method, and differences in variables were analyzed using the log-rank test. The results are presented as hazard ratios (HRs) and 95% confidence intervals (CIs). The characteristics and clinical outcomes were also assessed in subgroups that were stratified by the actionable mutation status.

All the statistical analyses were performed using the statistical software SPSS version 26.0 (SPSS, Inc., Chicago, IL, USA) and R version 4.2.2. Statistical tests were two-tailed, and a *P* < 0.05 indicated statistical significance.

## Results

### Patient characteristics

**Fig. 1:**
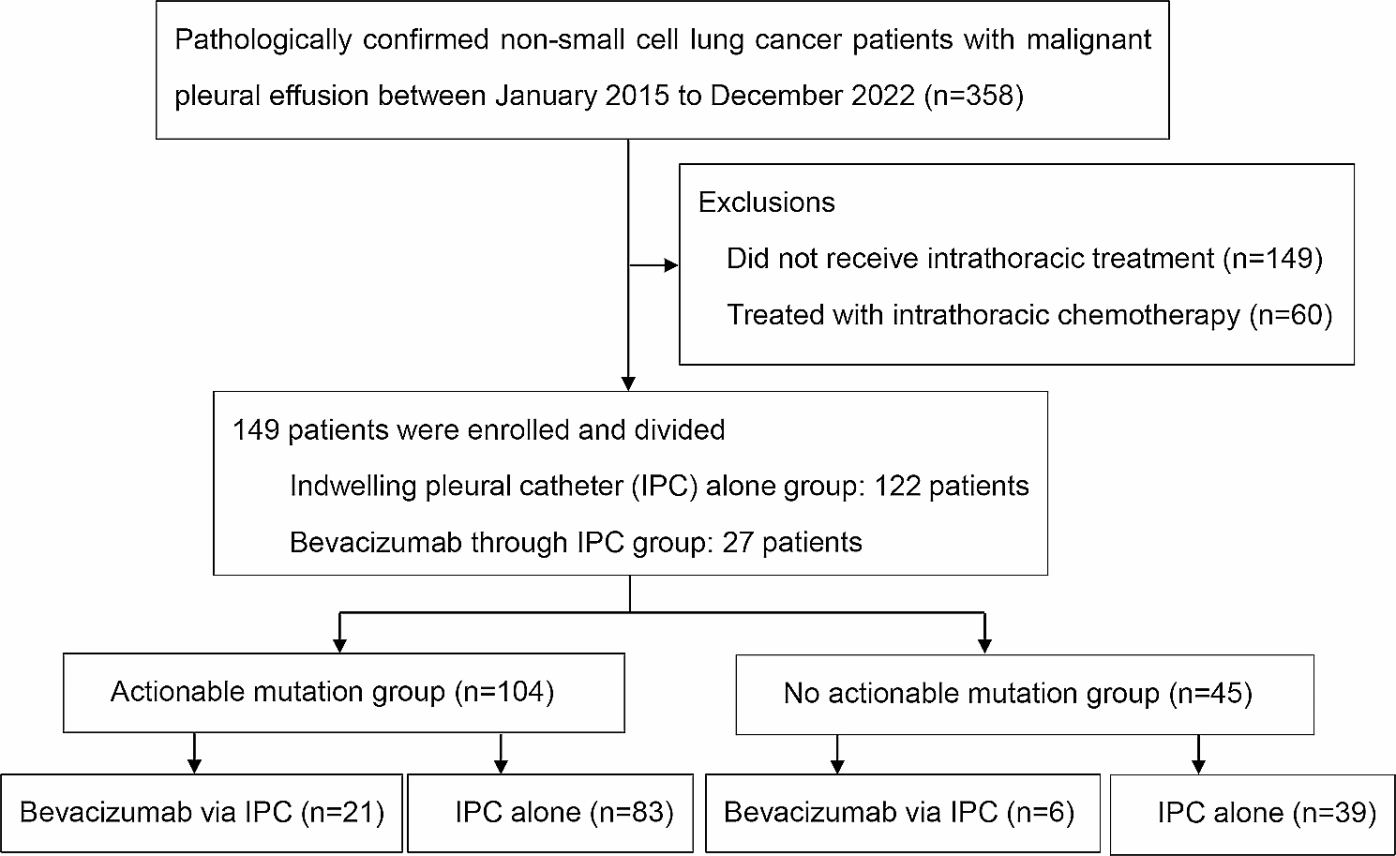
The workflow of patient selection

**Fig. 2:**
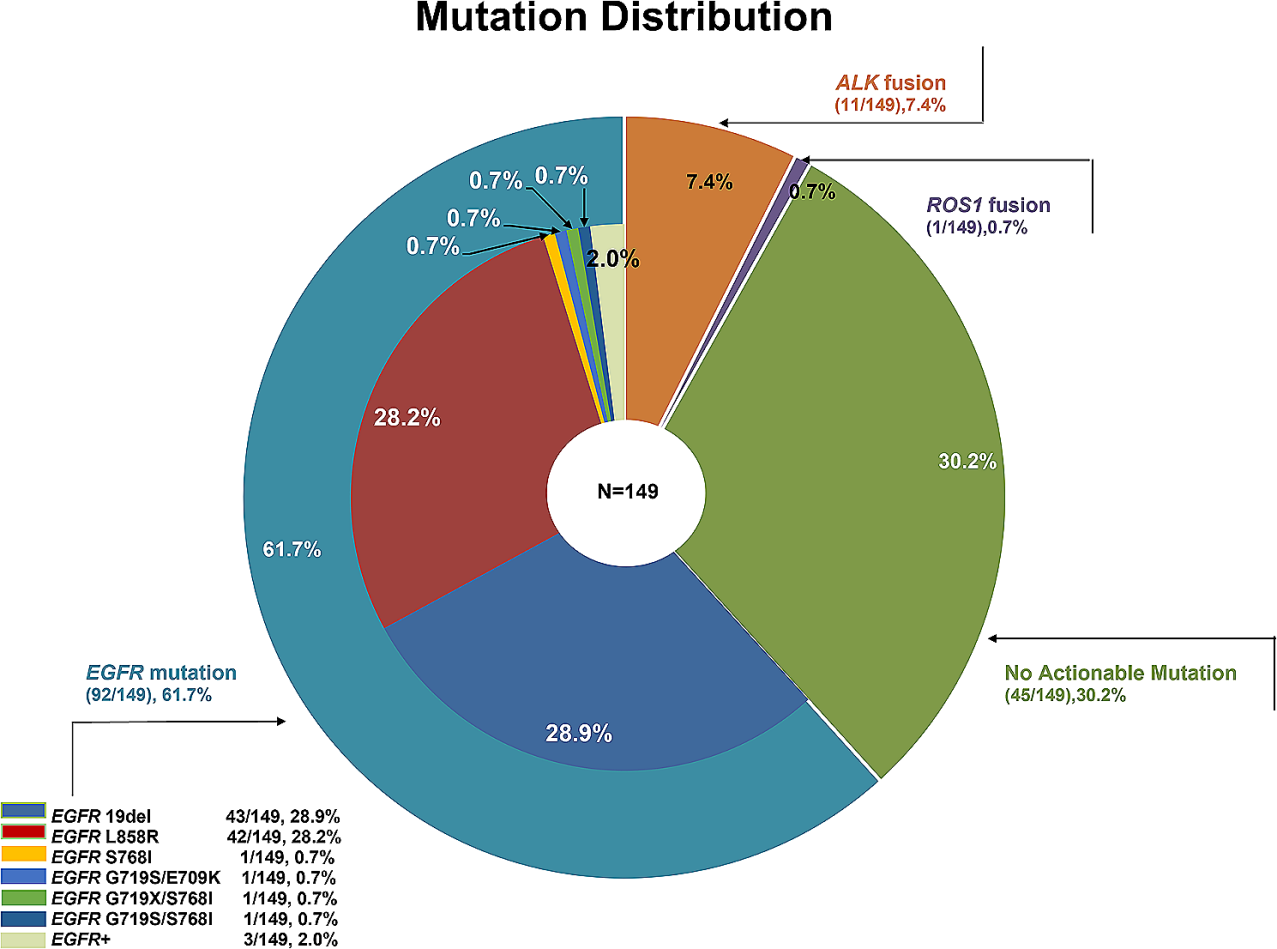
Mutation distribution of the 149 patients

A total of 358 consecutive patients with NSCLC and MPE were included; 149 asymptomatic patients who did not receive any intrathoracic treatment and 60 patients who were treated with intrathoracic chemotherapy were excluded. A total of 149 patients were included in our research. A total of 149 patients were included, and the median age was 60.3 years. Males and nonsmokers accounted for 48.3% and 65.8%, respectively. A total of 81.9% (122/149) of patients received an IPC alone, and 18.1% (27/149) received bevacizumab through an IPC (Fig. 1). A total of 69.8% (104/149) of patients harbored actionable mutations, including 92 *EGFR*-activating mutations, 11 *ALK* fusions, and 1 *ROS1* fusion (Fig. 2). There was no statistically significant difference in age, sex, history of smoking, Eastern Cooperative Oncology Group Performance Status (ECOG PS) score, clinical stage, pathological subtype, site of metastasis or systemic anticancer therapy between patients who received bevacizumab via an IPC and those who received an IPC alone (all *P* > 0.05) (Table 1).

**Table 1:** Baseline characteristics of patients with NSCLC and MPE who received bevacizumab through IPC or the IPC alone

Clinical characteristics	Bevacizumab through IPC (*N* = 27)	IPC (*N* = 122)	Total (*N* = 149)	*P*
Age (median [IQR])	61.62 [51.74, 68.89]	60.03 [50.95, 70.50]	60.30 [50.77, 70.45]	0.63
Age, *n* (%)				0.76
< 60	12 (44.4)	61 (50.0)	73 (49.0)	
≥ 60	15 (55.6)	61 (50.0)	76 (51.0)	
Gender, *n* (%)				0.51
Female	16 (59.3)	61 (50.0)	77 (51.7)	
Male	11 (40.7)	61 (50.0)	72 (48.3)	
Smoking, *n* (%)				0.15
Never	21 (77.8)	77 (63.1)	98 (65.8)	
Former/Current smoking	6 (22.2)	45 (36.9)	51 (34.2)	
Smoking index, *n* (%)				0.45
< 400	23 (85.2)	93 (76.2)	116 (77.9)	
≥ 400	4 (14.8)	29 (23.8)	33 (22.1)	
ECOG PS, *n* (%)				0.96
≥ 2	7 (25.9)	35 (28.7)	107 (71.8)	
0–1	20 (74.1)	87 (71.3)	42 (28.2)	
Pathology, *n* (%)				0.43
adenocarcinoma	24 (88.9)	115 (94.3)	139 (93.3)	
Squamous	3 (11.1)	6 (4.9)	9 (6.0)	
Others	0 (0.0)	1 (0.8)	1 (0.7)	
Stage, *n* (%)				0.35
IVA	20 (74.1)	76 (62.3)	96 (64.4)	
IVB	7 (25.9)	46 (37.7)	53 (35.6)	
Brain metastasis, *n* (%)				0.70
No	24 (88.9)	102 (83.6)	126 (84.6)	
Yes	3 (11.1)	20 (16.4)	23 (15.4)	
Live metastasis, *n* (%)				1.00
No	26 (96.3)	117 (95.9)	143 (96.0)	
Yes	1 (3.7)	5 (4.1)	6 (4.0)	
Bone metastasis, *n* (%)				1.00
No	19 (70.4)	87 (71.3)	106 (71.1)	
Yes	8 (29.6)	35 (28.7)	43 (28.9)	
Adrenal metastasis, *n* (%)				0.82
No	25 (92.6)	117 (95.9)	142 (95.3)	
Yes	2 (7.4)	5 (4.1)	7 (4.7)	
Pericardial metastasis, *n* (%)				1.00
No	26 (96.3)	117 (95.9)	143 (96.0)	
Yes	1 (3.7)	5 (4.1)	6 (4.0)	
Intrapulmonary metastasis, *n* (%)				0.89
No	20 (74.1)	86 (70.5)	106 (71.1)	
Yes	7 (25.9)	36 (29.5)	43 (28.9)	
MPE site, *n* (%)				0.90
Bilateral	3 (11.1)	12 (9.8)	15 (10.1)	
Left	14 (51.9)	59 (48.4)	73 (49.0)	
Right	10 (37.0)	51 (41.8)	61 (40.9)	
Systemic anticancer therapy, *n* (%)				0.13
Target therapy	20 (74.1)	80 (65.5)	100 (67.1)	
Target therapy + chemotherapy + antiangiogenic therapy	0	2 (1.6)	2 (1.3)	
Target therapy + antiangiogenic therapy	2 (7.4)	5 (4.1)	7 (4.7)	
Chemotherapy	0	13 (10.7)	13 (8.7)	
Chemotherapy + antiangiogenic therapy	2 (7.4)	3 (2.5)	5 (3.4)	
Chemotherapy + immunotherapy	2 (7.4)	16 (13.1)	18 (12.1)	
Immunotherapy	0	3 (2.5)	3 (2.0)	
Immunotherapy + antiangiogenic therapy	1 (3.7)	0	1 (0.7)	
Tumor-driver mutations status				0.32
Actionable mutation	21 (77.8)	83 (68.0)	104 (69.8)	
Without actionable mutation	6 (22.2)	39 (32.0)	45 (30.2)	

NSCLC: non-small cell lung cancer; MPE: Malignant pleural effusions; IPC: indwelling pleural catheter; IQR: interquartile range; ECOG PS: Eastern Cooperative Oncology Group Performance Status; Actionable mutation: patients with sensitizing *EGFR* mutation or *ALK/ROS1* fusion; Without actionable mutation: patients without sensitizing *EGFR* mutation or *ALK/ROS1* fusion

### The incidence of spontaneous pleurodesis at 3 months and 6 months

**Fig. 3:**
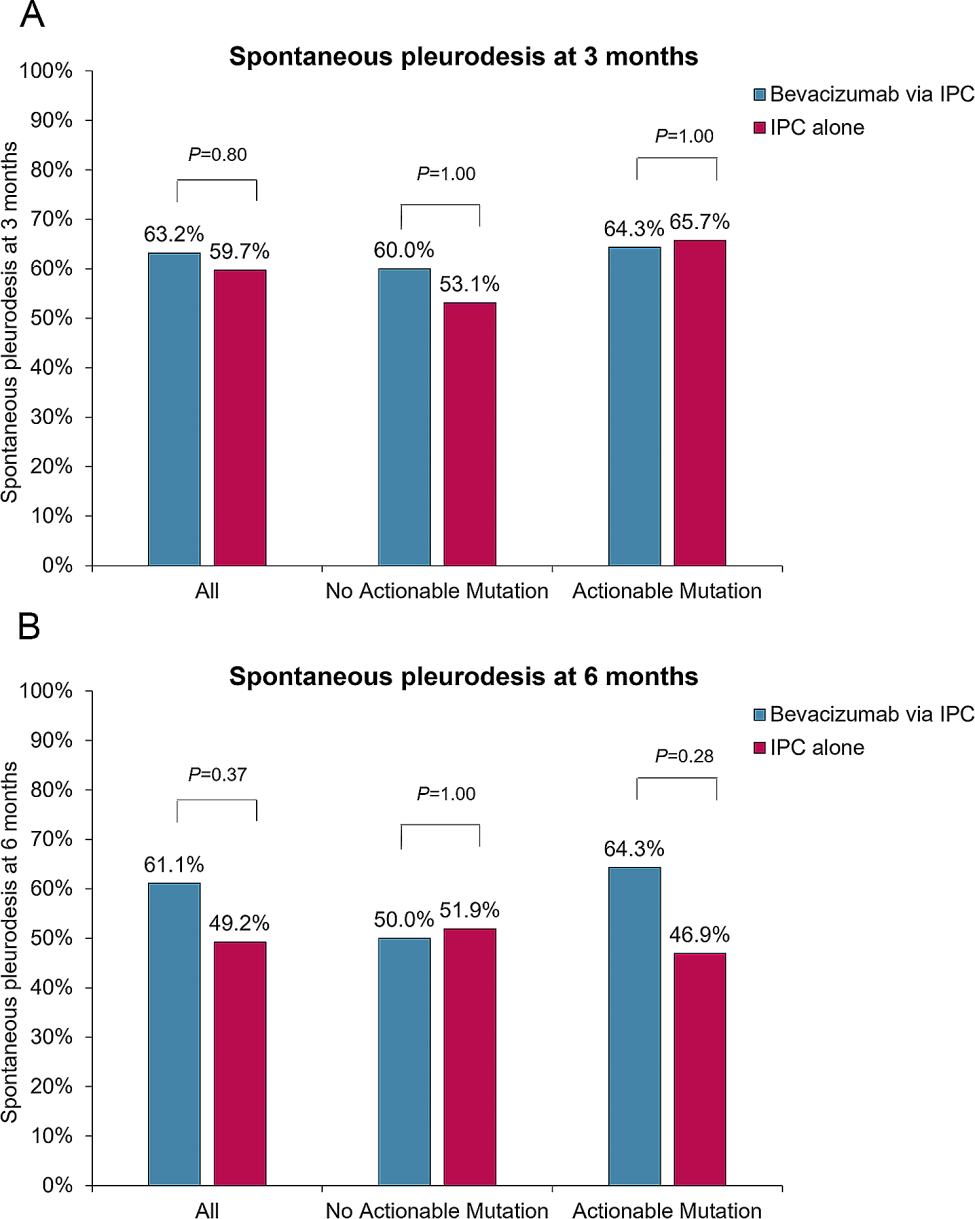
Comparison of the rates of spontaneous pleurodesis at 3 months (**A**) and at 6 months (**B**) between the bevacizumab through an IPC group and the IPC alone arm in the whole cohort, in patients without actionable mutations, and in patients with actionable mutations. IPC: indwelling pleural catheter

In the cohort, 86 patients and 77 patients were evaluated for spontaneous pleurodesis at 3 months and 6 months, respectively. In the whole cohort, spontaneous pleurodesis developed in 63.2% of those who received bevacizumab through an IPC at 3 months and developed in 59.7% of patients who were treated with an IPC alone at 3 months (*P* = 0.80) (Fig. 3A). In the subgroup of patients without actionable mutations, the rates of spontaneous pleurodesis at 3 months were 60.0% in the bevacizumab through an IPC arm and 53.1% in the IPC alone arm (*P* = 1.00) (Fig. 3A). However, in the subgroup of patients with actionable mutations, spontaneous pleurodesis was more common in those treated with bevacizumab through an IPC than in those treated with an IPC alone (64.3% vs. 46.9%, respectively) at 6 months but not at 3 months (64.3% vs. 65.7%, respectively) (Fig. 3A and B).

### Comparison of DCRs and OS

A total of 138 patients were evaluated for treatment efficacy in the cohort. The DCR was 95.9% in the bevacizumab through an IPC group and 85.0% in the IPC alone group (*P* = 0.20) (Additional file 1: Figure S1). No significant difference was found in the DCR between the patients in these two groups.

**Fig. 4:**
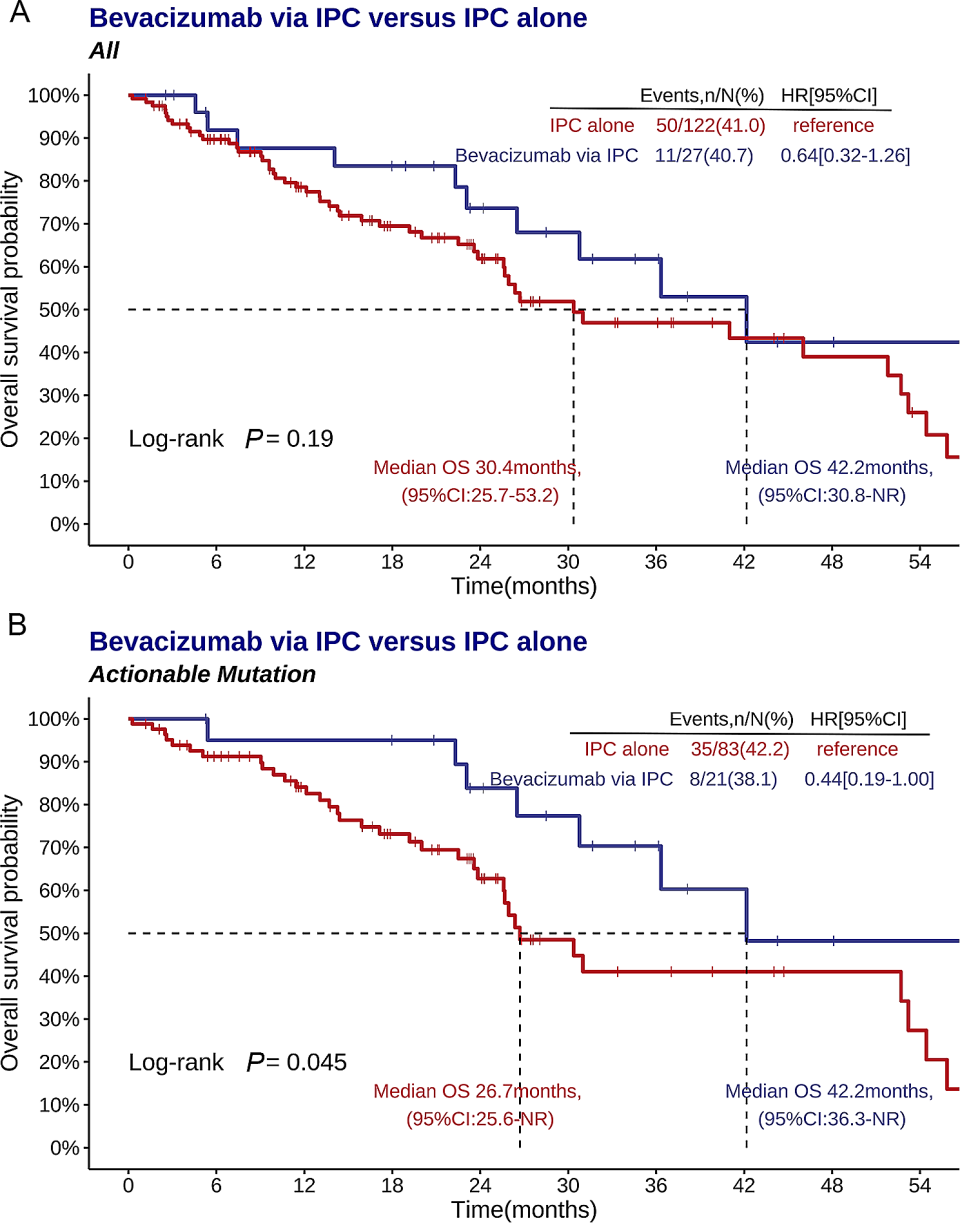
K‒M analyses of OS in the bevacizumab through an IPC group versus the IPC alone arm. OS in the whole cohort (**A**) and in patients with actionable mutations (**B**). OS: overall survival; IPC: indwelling pleural catheter

The median follow-up period was 17.5 (7.4–26.8) months. There was no difference in the median OS between the patients who received bevacizumab through an IPC or who an IPC alone (42.2 months [95% CI: 30.8 to not reached (NR)] and 30.4 months [95% CI: 25.7 to 53.2], respectively) (HR = 0.64; 95% CI: 0.32 to 1.26; *P* = 0.19) (Fig. 4A).

### Subgroup analyses of patients without actionable mutations

Forty-five patients were divided into subgroups without actionable mutations. The bevacizumab through an IPC group and the IPC alone group included 6 and 39 patients, respectively (Fig. 1). The baseline clinical characteristics were well-balanced between the two groups (Additional file 2: Table S1). The DCR was 80.0% in the bevacizumab through the IPC group and 77.1% in the IPC alone group (*P* = 1.00) (Additional file 1: Figure S1).

The median OS was similar in the bevacizumab through an IPC group compared with the IPC alone group (10.8 months (95% CI: 4.6 to NR) and 41.0 months (95% CI: 13.0 to NR)) (HR = 2.08; 95% CI: 0.59 to 7.40; *P* = 0.24) (Additional file 1: Figure S2).

### Subgroup analyses of patients with actionable mutations

Among the 104 patients with actionable mutations, 21 patients were treated with bevacizumab through an IPC, and 83 patients received an IPC alone (Fig. 1). The baseline clinical characteristics were well balanced between the treatment arms (Additional file 2: Table S2). No significant differences were observed in the DCR between the bevacizumab through an IPC group and the IPC alone group (100.0% vs. 88.6%, *P* = 0.20; Additional file 1: Figure S1).

**Fig. 5:**
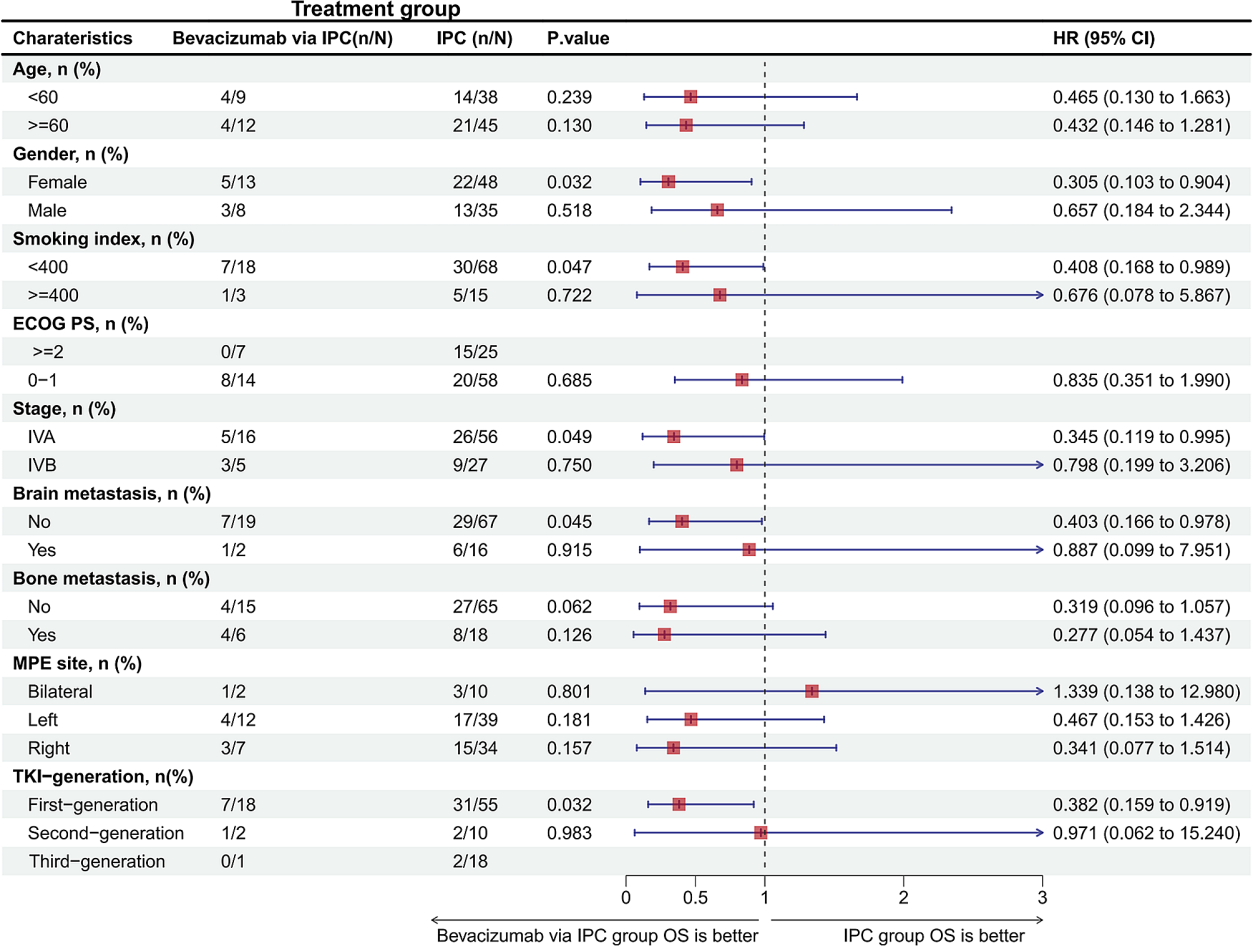
Subgroup analysis of overall survival of patients with actionable mutations according to baseline characteristics CI: confidence interval; HR: hazard ratio; ECOG PS: Eastern Cooperative Oncology Group Performance Status; TKI: tyrosine kinase inhibitor; MPE: malignant pleural effusion

However, the median OS was significantly longer in patients who received bevacizumab via an IPC than in those treated with an IPC alone (42.2 months (95% CI: 36.3 to NR) vs. 26.7 months (95% CI: 25.6 to NR), respectively) (HR = 0.44; 95% CI: 0.19 to 1.00; *P* = 0.045; Fig. 4B). Additionally, the subgroup analyses revealed that compared to the IPC alone group, there was a consistent improvement in OS with bevacizumab through an IPC across the majority of the clinical subgroups, except for patients who had bilateral MPE, in whom bevacizumab through an IPC did not improve OS (Fig. 5).

### Adverse events

Among patients with actionable mutations, 3 of the 21 patients in the bevacizumab through an IPC group experienced any adverse events, whereas 9 of the 83 patients in the IPC group experienced adverse events. For patients without actionable mutations, 1 of the 6 patients experienced any adverse events in the bevacizumab through an IPC group, and 5 of the 39 patients in the IPC group experienced adverse events. Pain, catheter blockage and fever were the most common adverse events associated with bevacizumab in the IPC group (Additional file 2: Table S3).

No significant difference between the bevacizumab through an IPC group and the IPC group was detected in the number of participants who had adverse events, either in patients with actionable mutations (14.3% vs. 8.4%; *P* = 0.42) or in patients with no actionable mutations (16.7% vs. 12.8%; *P* = 1.00) (Additional file 1: Figure S3).

## Discussion

To the best of our knowledge, we first assessed the impact of bevacizumab through an IPC on the prognosis of patients with NSCLC and MPE. This study showed that the efficacy of bevacizumab through an IPC was similar to that of IPC alone in patients without actionable mutations. However, the incidence of spontaneous pleurodesis during the first 6 months was greater, and the median OS was significantly longer with bevacizumab through an IPC than with IPC alone in patients with actionable mutations. Additionally, the subgroup analyses revealed a consistent improvement in OS with bevacizumab through an IPC across the majority of clinical subgroups, and the safety of bevacizumab through an IPC was comparable to that of IPC alone. As shown in previous studies, intrapleural therapy with bevacizumab was found to be safe for managing NSCLC-mediated MPE [10, 26]. Together with these results, it is suggested that the option of intrapleural use of bevacizumab through an IPC be considered for NSCLC patients with symptomatic MPE and actionable mutations, except for those patients with bilateral MPE.

As observed in our study, bevacizumab through an IPC significantly prolonged OS when compared with IPC alone in patients with an actionable mutation. These results are in line with those of Xiang Z et al. [27], who revealed that intrapleural antiangiogenic therapy plus cisplatin significantly prolonged the OS of Lewis lung cancer (LLC)-induced MPE model mice by reducing MPE. We considered that the favorable OS in NSCLC patients with MPE and an actionable mutation was attributed to bevacizumab administered through an IPC but was not related to systemic anticancer therapy. An observational study showed that systemic anticancer treatment was not related independently to MPE resolution in pharmacologically sensitive tumors [28]. Similarly, the phase 3 trials NEJ026 [29] and ATLAS [30] revealed that intravenous bevacizumab plus erlotinib did not prolong OS compared with erlotinib alone in patients with metastatic *EGFR*-mutant NSCLC. A randomized clinical study demonstrated that intrapleural bevacizumab therapy was more effective than intravenous infusions of bevacizumab in patients with NSCLC and MPE [12]. Furthermore, there was no statistically significant difference in the systemic anticancer therapy between patients who received bevacizumab via an IPC and those who received IPC alone.

Although intrapleural administration of bevacizumab is an effective treatment strategy for NSCLC patients with MPE [13, 31, 32], whether specific mutations affect the efficacy of bevacizumab has not been reported. Interestingly, we found that only patients with actionable mutations could benefit from bevacizumab through an IPC. Stratified analysis of the phase 3 IMpower150 study [33] demonstrated that the median OS in patients with *EGFR* mutations who received bevacizumab plus carboplatin and paclitaxel was longer than that in patients with wild-type EGFR who received the same treatment regimen [34]. Additionally, Chen et al. [35] reported that gefitinib plus bevacizumab inhibited MPE-induced endothelial angiogenesis better than gefitinib alone in the *EGFR* mutation subgroup. Thus, intracavitary infusion of bevacizumab may be more beneficial in patients with tumor driver mutations, which is consistent with our findings.

Almost all approved antiangiogenic drugs target the VEGF pathway; thus, the efficacy of bevacizumab is likely related to the level of VEGF in MPE [31]. As demonstrated by Du N et al. [10], intrapleural bevacizumab therapy was more effective in MPE patients with high VEGF expression than in those patients who were VEGF negative. Interestingly, studies have suggested that *EGFR* can upregulate VEGF expression via MAPK and PI3K signaling [36]. Similarly, Watanabe H et al. [37] showed that the protein levels of VEGF-A, a member of the VEGF family [38], were elevated in NSCLC cell lines harboring *EGFR*, *ALK*, or *ROS1* alterations. Therefore, it can be inferred that patients with MPE and an actionable mutation may have high VEGF expression and thus are most likely to benefit from intrapleural administration of bevacizumab.

Our study has several limitations. Because this was a retrospective study with a relatively small sample size, especially for patients without actionable mutations, the results should be interpreted cautiously, and further validation is needed in a larger population. However, the results of our study are consistent with those of published phase III clinical studies of patients with NSCLC, suggesting that antiangiogenic therapy may improve the clinical outcome of NSCLC patients with actionable mutations [33, 34, 39]. Additionally, we did not compare the VEGF levels in MPE patients with and without actionable mutations because of the limited number of MPE samples.

## Conclusions

Bevacizumab through an IPC significantly improved the prognosis of NSCLC patients with MPE and actionable mutations, and the safety of bevacizumab through an IPC was comparable to that of an IPC alone. However, patients without actionable mutations do not benefit from bevacizumab through an IPC. Therefore, an IPC alone is likely to remain the standard treatment for patients without actionable mutations to avoid additional adverse effects of intracavitary infusions of bevacizumab.

### Electronic supplementary material

Below is the link to the electronic supplementary material.

Supplementary Material 1

Supplementary Material 2

## Data Availability

All data generated or analyzed during this study are included in this published article. The data that support the findings of this study are available from the corresponding author upon reasonable request.

## Abbreviations

CI: Confidence interval
CR: Complete response
CT: Computed tomography
DCR: Disease control rate
ECOG PS: Eastern Cooperative Oncology Group Performance Status
HRs: Hazard ratios
IPC: Indwelling pleural catheter
IQR: Interquartile range
MPE: Malignant pleural effusion
MRI: Magnetic resonance imaging
NGS: Next-generation sequencing
NSCLC: Non small cell lung cancer
OS: Overall survival
PR: Partial response
SD: Stable disease
TKIs: Tyrosine kinase inhibitors

## References

[1] Jacobs B, Sheikh G, Youness HA, Keddissi JI, Abdo T. Diagnosis and management of malignant pleural effusion: a decade in review. Diagnostics (Basel Switzerland). 2022;12(4). 10.3390/diagnostics12041016 10.3390/diagnostics12041016 PMC9030780 35454064

[2] Taghizadeh N, Fortin M, Tremblay A (2017).

[3] Tian P, Qiu R, Wang M, Xu S, Cao L, Yang P, Li W (2021). Prevalence, causes, and Health Care Burden of Pleural effusions among hospitalized adults in China. JAMA Netw open.

[4] Bashour SI, Mankidy BJ, Lazarus DR (2022). Update on the diagnosis and management of malignant pleural effusions. Respir Med.

[5] Zhao Y, Yu L, Wang L, Wu Y, Chen H, Wang Q, Wu Y (2022). Current status of and progress in the treatment of malignant pleural effusion of lung cancer. Front Oncol.

[6] Schwalk AJ, Ost DE, Saltijeral SN, De La Garza H, Casal RF, Jimenez CA, Eapen GA, Lewis J, Rinsurongkawong W, Rinsurongkawong V (2021). Risk factors for and Time to recurrence of symptomatic malignant pleural effusion in patients with metastatic non-small cell lung Cancer with EGFR or ALK mutations. Chest.

[7] Bibby AC, Dorn P, Psallidas I, Porcel JM, Janssen J, Froudarakis M, Subotic D, Astoul P, Licht P, Schmid R, et al. ERS/EACTS statement on the management of malignant pleural effusions. Eur Respir J. 2018;52(1). 10.1183/13993003.00349-2018 10.1183/13993003.00349-2018 30054348

[8] Roberts ME, Rahman NM, Maskell NA, Bibby AC, Blyth KG, Corcoran JP, Edey A, Evison M, de Fonseka D, Hallifax R (2023). British thoracic Society Guideline for pleural disease. Thorax.

[9] Feller-Kopman DJ, Reddy CB, DeCamp MM, Diekemper RL, Gould MK, Henry T, Iyer NP, Lee YCG, Lewis SZ, Maskell NA (2018). Management of malignant pleural effusions. An official ATS/STS/STR clinical practice Guideline. Am J Respir Crit Care Med.

[10] Du N, Li X, Li F, Zhao H, Fan Z, Ma J, Fu Y, Kang H (2013). Intrapleural combination therapy with bevacizumab and cisplatin for non-small cell lung cancer–mediated malignant pleural effusion. Oncol Rep.

[11] Sack U, Hoffmann M, Zhao XJ, Chan KS, Hui DS, Gosse H, Engelmann L, Schauer J, Emmrich F, Hoheisel G (2005). Vascular endothelial growth factor in pleural effusions of different origin. Eur Respir J.

[12] Nie K, Zhang Z, You Y, Zhuang X, Zhang C, Ji Y (2020). A randomized clinical study to compare intrapleural infusion with intravenous infusion of bevacizumab in the management of malignant pleural effusion in patients with non-small-cell lung cancer. Thorac cancer.

[13] Di W, Yue C, Ziran Z, Jie Z, Jun N, Ling D, Weiheng H, Xiaoling C, Xiangjuan M, Guangming T (2022). A phase II study of bevacizumab in non-squamous, non-small-cell lung cancer patients with malignant pleural effusion. Future Oncol.

[14] Zhou Z, Li H, Hu D, Xie L (2021). Clinical efficacy of bevacizumab combined with cisplatin in the treatment of malignant pleural effusion and ascites caused by lung cancer: a randomized trial. Annals of Palliative Medicine.

[15] Saito H, Fukuhara T, Furuya N, Watanabe K, Sugawara S, Iwasawa S, Tsunezuka Y, Yamaguchi O, Okada M, Yoshimori K (2019). Erlotinib plus Bevacizumab versus erlotinib alone in patients with EGFR-positive advanced non-squamous non-small-cell lung cancer (NEJ026): interim analysis of an open-label, randomised, multicentre, phase 3 trial. Lancet Oncol.

[16] Zhou Q, Xu CR, Cheng Y, Liu YP, Chen GY, Cui JW, Yang N, Song Y, Li XL, Lu S (2021). Bevacizumab plus Erlotinib in Chinese patients with untreated, EGFR-mutated, advanced NSCLC (ARTEMIS-CTONG1509): a multicenter phase 3 study. Cancer Cell.

[17] Travis WD, Brambilla E, Nicholson AG, Yatabe Y, Austin JHM, Beasley MB, Chirieac LR, Dacic S, Duhig E, Flieder DB (2015). The 2015 World Health Organization Classification of Lung Tumors: impact of genetic, clinical and radiologic advances since the 2004 classification. J Thorac Oncol.

[18] Chansky K, Detterbeck FC, Nicholson AG, Rusch VW, Vallières E, Groome P, Kennedy C, Krasnik M, Peake M, Shemanski L (2017). The IASLC Lung Cancer Staging Project: external validation of the revision of the TNM Stage groupings in the Eighth Edition of the TNM classification of Lung Cancer. J Thorac Oncol.

[19] Ettinger DS, Wood DE, Aisner DL, Akerley W, Bauman JR, Bharat A, Bruno DS, Chang JY, Chirieac LR, D’Amico TA (2021). NCCN guidelines Insights: Non-small Cell Lung Cancer, Version 2.2021. J Natl Compr Canc Netw.

[20] Planchard D, Popat S, Kerr K, Novello S, Smit EF, Faivre-Finn C, Mok TS, Reck M, Van Schil PE, Hellmann MD (2018). Metastatic non-small cell lung cancer: ESMO Clinical Practice guidelines for diagnosis, treatment and follow-up. Ann Oncol.

[21] Chaddha U, Agrawal A, Bhavani SV, Sivertsen K, Donington DJ, Ferguson MK, Murgu S (2021). Thoracic ultrasound as a predictor of pleurodesis success at the time of indwelling pleural catheter removal. Respirology.

[22] Suzuki K, Servais EL, Rizk NP, Solomon SB, Sima CS, Park BJ, Kachala SS, Zlobinsky M, Rusch VW, Adusumilli PS (2011). Palliation and pleurodesis in malignant pleural effusion: the role for tunneled pleural catheters. J Thorac Oncol.

[23] Schwartz LH, Litière S, de Vries E, Ford R, Gwyther S, Mandrekar S, Shankar L, Bogaerts J, Chen A, Dancey J (2016). RECIST 1.1-Update and clarification: from the RECIST committee. Eur J Cancer.

[24] Soo RA, Han JY, Dafni U, Cho BC, Yeo CM, Nadal E, Carcereny E, de Castro J, Sala MA, Bernabé R (2022). A randomised phase II study of osimertinib and bevacizumab versus osimertinib alone as second-line targeted treatment in advanced NSCLC with confirmed EGFR and acquired T790M mutations: the European thoracic oncology platform (ETOP 10–16) BOOSTER trial. Ann Oncol.

[25] Shi Y, Wu L, Yu X, Xing P, Wang Y, Zhou J, Wang A, Shi J, Hu Y, Wang Z et al. Sintilimab versus Docetaxel as second-line treatment in advanced or metastatic squamous non-small-cell lung cancer: an open-label, randomized controlled phase 3 trial (ORIENT-3). Cancer communications (London, England). 2022; 42(12):1314–30. 10.1002/cac2.12385 10.1002/cac2.12385 PMC9759762 36336841

[26] Song X, Chen D, Guo J, Kong L, Wang H, Wang Z (2018). Better efficacy of intrapleural infusion of bevacizumab with pemetrexed for malignant pleural effusion mediated from nonsquamous non-small cell lung cancer. Onco Targets Ther.

[27] Xiang Z, Deng X, He W, Yang Q, Ni L, Dehghan Shasaltaneh M, Maghsoudloo M, Yang G, Wu J, Imani S (2022). Treatment of malignant pleural effusion in non-small cell lung cancer with VEGF-directed therapy. Ann Med.

[28] Holling N, Patole S, Medford ARL, Maskell NA, Bibby AC (2021). Is systemic Anticancer Therapy Associated with higher rates of malignant pleural effusion control in people with pharmacologically sensitive tumors? A retrospective analysis of prospectively Collected Data. Chest.

[29] Kawashima Y, Fukuhara T, Saito H, Furuya N, Watanabe K, Sugawara S, Iwasawa S, Tsunezuka Y, Yamaguchi O, Okada M (2022). Bevacizumab plus Erlotinib versus erlotinib alone in Japanese patients with advanced, metastatic, EGFR-mutant non-small-cell lung cancer (NEJ026): overall survival analysis of an open-label, randomised, multicentre, phase 3 trial. The Lancet Respiratory Medicine.

[30] Johnson BE, Kabbinavar F, Fehrenbacher L, Hainsworth J, Kasubhai S, Kressel B, Lin CY, Marsland T, Patel T, Polikoff J (2013). ATLAS: randomized, double-blind, placebo-controlled, phase IIIB trial comparing bevacizumab therapy with or without erlotinib, after completion of chemotherapy, with bevacizumab for first-line treatment of advanced non-small-cell lung cancer. J Clin Oncol.

[31] Jayson GC, Kerbel R, Ellis LM, Harris AL (2016). Antiangiogenic therapy in oncology: current status and future directions. Lancet.

[32] Tamiya M, Tamiya A, Suzuki H, Taniguchi Y, Katayama K, Minomo S, Nakao K, Takeuchi N, Matsuda Y, Naito Y (2021). Phase 2 study of bevacizumab plus carboplatin/nab-paclitaxel followed by bevacizumab plus nab-paclitaxel for non-squamous non-small cell lung cancer with malignant pleural effusion. Invest New Drugs.

[33] Socinski MA, Nishio M, Jotte RM, Cappuzzo F, Orlandi F, Stroyakovskiy D, Nogami N, Rodríguez-Abreu D, Moro-Sibilot D, Thomas CA (2021). IMpower150 final overall survival analyses for Atezolizumab Plus Bevacizumab and Chemotherapy in First-Line Metastatic Nonsquamous NSCLC. J Thorac Oncol.

[34] Nogami N, Barlesi F, Socinski MA, Reck M, Thomas CA, Cappuzzo F, Mok TSK, Finley G, Aerts JG, Orlandi F (2022). IMpower150 final exploratory analyses for Atezolizumab Plus Bevacizumab and Chemotherapy in Key NSCLC patient subgroups with EGFR mutations or metastases in the liver or brain. J Thorac Oncol.

[35] Chen WT, Lin YH, Changchien CY, Chen Y, Chang HH, Tsai WC, Tsai HC, Wang CY, Shen MS, Cheng LT, et al. Concurrent blockade of endothelial EGFR and VEGF signaling on malignant associated pleural fluid induced angiogenesis: from clinic to bench. Biomedicines. 2021;9(10). 10.3390/biomedicines9101327 10.3390/biomedicines9101327 PMC8533568 34680445

[36] Larsen AK, Ouaret D, El Ouadrani K, Petitprez A (2011). Targeting EGFR and VEGF(R) pathway cross-talk in tumor survival and angiogenesis. Pharmacol Ther.

[37] Watanabe H, Ichihara E, Kayatani H, Makimoto G, Ninomiya K, Nishii K, Higo H, Ando C, Okawa S, Nakasuka T (2021). VEGFR2 blockade augments the effects of tyrosine kinase inhibitors by inhibiting angiogenesis and oncogenic signaling in oncogene-driven non-small-cell lung cancers. Cancer Sci.

[38] Le X, Nilsson M, Goldman J, Reck M, Nakagawa K, Kato T, Ares LP, Frimodt-Moller B, Wolff K, Visseren-Grul C (2021). Dual EGFR-VEGF pathway inhibition: a promising strategy for patients with EGFR-Mutant NSCLC. J Thorac Oncol.

[39] West H, McCleod M, Hussein M, Morabito A, Rittmeyer A, Conter HJ, Kopp HG, Daniel D, McCune S, Mekhail T (2019). Atezolizumab in combination with carboplatin plus nab-paclitaxel chemotherapy compared with chemotherapy alone as first-line treatment for metastatic non-squamous non-small-cell lung cancer (IMpower130): a multicentre, randomised, open-label, phase 3 trial. Lancet Oncol.

