# Supplementary material for: Efficacy of bevacizumab through an indwelling pleural catheter in non-small cell lung cancer patients with symptomatic malignant pleural effusion
Source: BMC Pulm Med. 2024 Feb 16;24:89. doi: 10.1186/s12890-024-02886-1 (PMC10874116; doi:10.1186/s12890-024-02886-1)
Supplement: Supplementary file 1 — Supplementary Material 1 [file 12890_2024_2886_MOESM1_ESM.docx]

**Additional file 1**

**
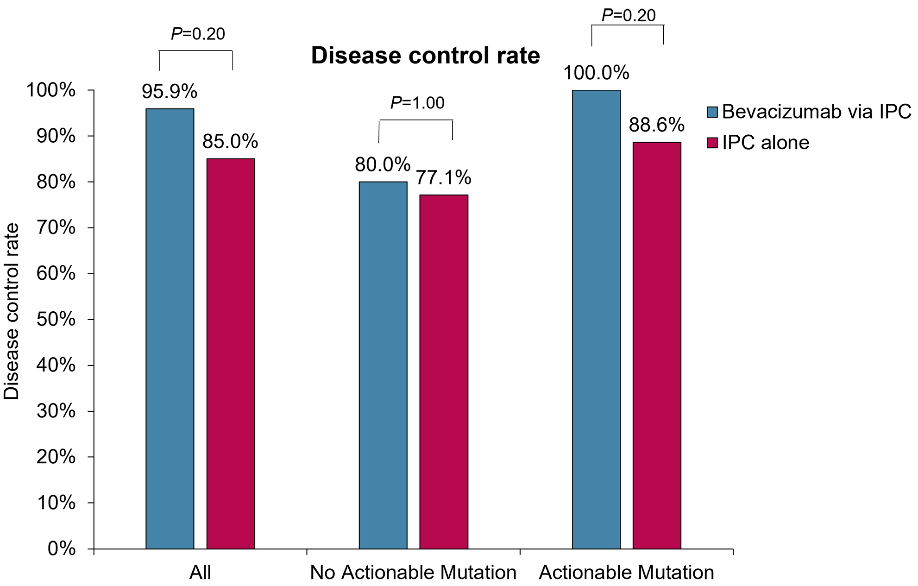
**

**Figures S1** The bar chart shows DCR. Comparison of the DCR between bevacizumab through an IPC group and the IPC alone arm in the whole cohort (the bar chart on the left), in patients without actionable mutations (the bar chart in the middle), and in patients with actionable mutations (the bar chart on the left). IPC: indwelling pleural catheter; DCR: disease control rate.


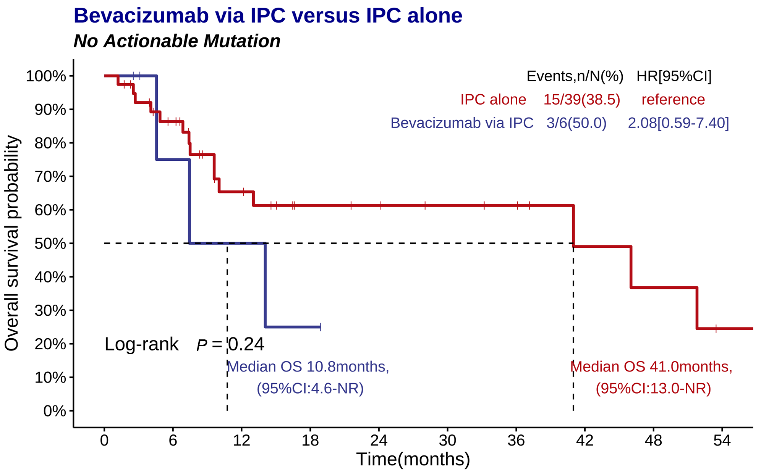


**Figures S2** The OS of patients without actionable mutations. K‒M analyses of OS in the bevacizumab through an IPC group (the blue line) versus the IPC alone arm (the red line) in patients without actionable mutations. OS: overall survival; IPC: indwelling pleural catheter.

**
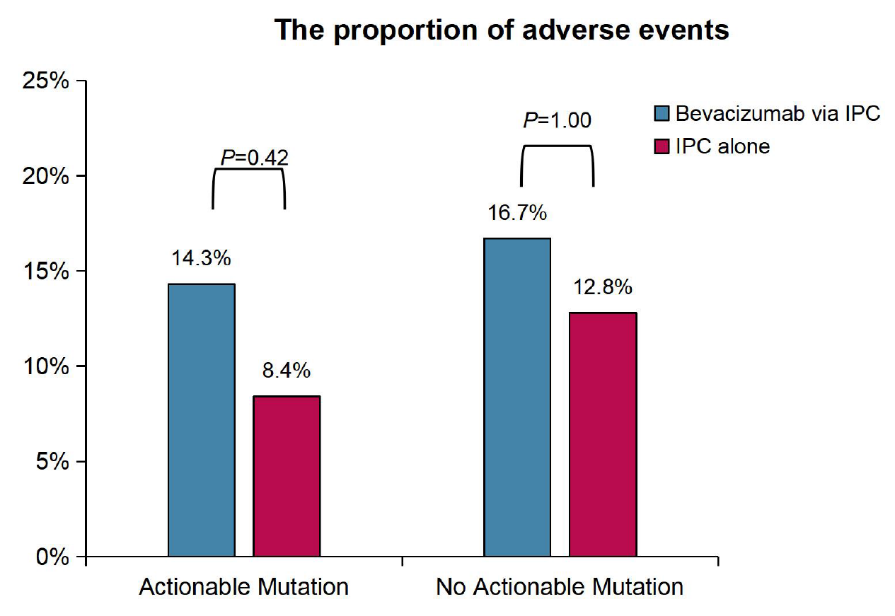
**

**Figures S3** The bar chart shows the proportion of adverse events. The proportion of adverse events in patients with actionable mutations (the bar chart on the left) or without actionable mutations (the bar chart on the right) who received bevacizumab through an IPC or who had an IPC alone.
