# Supplementary material for: Efficacy of bevacizumab through an indwelling pleural catheter in non-small cell lung cancer patients with symptomatic malignant pleural effusion
Source: BMC Pulm Med. 2024 Feb 16;24:89. doi: 10.1186/s12890-024-02886-1 (PMC10874116; doi:10.1186/s12890-024-02886-1)
Supplement: Supplementary file 2 — Supplementary Material 2 [file 12890_2024_2886_MOESM2_ESM.docx]

**Table S1 Baseline characteristics of patients without actionable mutation who received bevacizumab through IPC or the IPC alone**

| Clinical characteristics | Bevacizumab through IPC (n=6) | IPC (n=39) | P |
| --- | --- | --- | --- |
| Age (median [IQR]) | 58.70  [48.32, 75.77] | 56.80  [48.85, 66.83] | 0.57 |
| Age, n (%) |  |  | 1 |
| <60 | 3 (50.0) | 23 (59.0) |  |
| ≥60 | 3 (50.0) | 16 (41.0) |  |
| Gender, n (%) |  |  | 0.74 |
| Female | 3 (50.0) | 13 (33.3) |  |
| Male | 3 (50.0) | 26 (66.7) |  |
| Smoking index, n (%) |  |  | 0.64 |
| <400 | 5 (83.3) | 25 (64.1) |  |
| ≥400 | 1 (16.7) | 14 (35.9) |  |
| ECOG PS, n (%) |  |  | 0.38 |
| ≥2 | 0 (0.0) | 10 (25.6) |  |
| 0-1 | 6 (100.0) | 29 (74.4) |  |
| Pathology, n (%) |  |  | 0.08 |
| adenocarcinoma | 3 (50.0) | 33 (84.6) |  |
| Squamous | 3 (50.0) | 5 (12.8) |  |
| Other | 0 (0.0) | 1 (2.6) |  |
| Stage, n (%) |  |  | 0.79 |
| IVA | 4 (66.7) | 20 (51.3) |  |
| IVB | 2 (33.3) | 19 (48.7) |  |
| Brain metastasis, n (%) |  |  | 1 |
| No | 5 (83.3) | 35 (89.7) |  |
| Yes | 1 (16.7) | 4 (10.3) |  |
| Live metastasis, n (%) |  |  | 1 |
| No | 5 (83.3) | 35 (89.7) |  |
| Yes | 1 (16.7) | 4 (10.3) |  |
| Bone metastasis, n (%) |  |  | 0.98 |
| No | 4 (66.7) | 22 (56.4) |  |
| Yes | 2 (33.3) | 17 (43.6) |  |
| Adrenal metastasis, n (%) |  |  | 0.05 |
| No | 4 (66.7) | 38 (97.4) |  |
| Yes | 2 (33.3) | 1 (2.6) |  |
| Intrapulmonary metastasis, n (%) |  |  | 0.64 |
| No | 5 (83.3) | 25 (64.1) |  |
| Yes | 1 (16.7) | 14 (35.9) |  |
| MPE site, n (%) |  |  | 0.49 |
| Bilateral | 1 (16.7) | 2 (5.1) |  |
| Left | 2 (33.3) | 20 (51.3) |  |
| Right | 3 (50.0) | 17 (43.6) |  |

MPE: Malignant pleural effusions; IPC: indwelling pleural catheter; IQR: interquartile range; ECOG PS: Eastern Cooperative Oncology Group Performance Status; Actionable mutation: patients with sensitizing *EGFR* mutation or *ALK/ROS1* fusion.

**Table S2 Baseline characteristics of patients with actionable mutation who received bevacizumab through IPC or the IPC alone**

| Clinical characteristics | Bevacizumab  through IPC (n=21) | IPC (n=83) | P |
| --- | --- | --- | --- |
| Age (median [IQR]) | 61.62 [54.33, 66.92] | 63.23 [52.73, 72.03] | 0.91 |
| Age, n (%) |  |  | 1 |
| <60 | 9 (42.9) | 38 (45.8) |  |
| ≥60 | 12 (57.1) | 45 (54.2) |  |
| Gender, n (%) |  |  | 0.93 |
| Female | 13 (61.9) | 48 (57.8) |  |
| Male | 8 (38.1) | 35 (42.2) |  |
| Smoking index, n (%) |  |  | 0.93 |
| <400 | 18 (85.7) | 68 (81.9) |  |
| ≥400 | 3 (14.3) | 15 (18.1) |  |
| ECOG PS, n (%) |  |  | 0.98 |
| ≥2 | 7 (33.3) | 25 (30.1) |  |
| 0-1 | 14 (66.7) | 58 (69.9) |  |
| Pathology, n (%) |  |  | 1 |
| adenocarcinoma | 21 (100.0) | 82 (98.8) |  |
| Squamous | 0 (0.0) | 1 (1.2) |  |
| Stage, n (%) |  |  | 0.61 |
| IVA | 16 (76.2) | 56 (67.5) |  |
| IVB | 5 (23.8) | 27 (32.5) |  |
| Brain metastasis, n (%) |  |  | 0.46 |
| No | 19 (90.5) | 67 (80.7) |  |
| Yes | 2 (9.5) | 16 (19.3) |  |
| Live metastasis, n (%) |  |  | 1 |
| No | 21 (100.0) | 82 (98.8) |  |
| Yes | 0 (0.0) | 1 (1.2) |  |
| Bone metastasis, n (%) |  |  | 0.71 |
| No | 15 (71.4) | 65 (78.3) |  |
| Yes | 6 (28.6) | 18 (21.7) |  |
| Adrenal metastasis, n (%) |  |  | 0.7 |
| No | 21 (100.0) | 79 (95.2) |  |
| Yes | 0 (0.0) | 4 (4.8) |  |
| Intrapulmonary metastasis,  n (%) |  |  | 1 |
| No | 15 (71.4) | 61 (73.5) |  |
| Yes | 6 (28.6) | 22 (26.5) |  |
| MPE site, n (%) |  |  | 0.71 |
| Bilateral | 2 (9.5) | 10 (12.0) |  |
| Left | 12 (57.1) | 39 (47.0) |  |
| Right | 7 (33.3) | 34 (41.0) |  |
| Systemic anticancer therapy, n (%) |  |  |  |
| Target therapy | 19 (90.5) | 76 (91.6) | 0.67 |
| Target therapy  + antiangiogenic therapy | 2 (9.5) | 5 (6.0) |  |
| Target therapy  + chemotherapy  + antiangiogenic therapy | 0 (0.0) | 2 (2.4) |  |
| TKI-generation, n (%) |  |  |  |
| First-generation | 18 (85.7) | 55 (66.3) | 0.16 |
| Second-generation | 2 (9.5) | 10 (12.0) |  |
| Third-generation | 1 (4.8) | 18 (21.7) |  |

MPE: Malignant pleural effusions; IPC: indwelling pleural catheter; IQR: interquartile range; ECOG PS: Eastern Cooperative Oncology Group Performance Status; Actionable mutation: patients with sensitizing *EGFR* mutation or *ALK/ROS1* fusion.

**Table S3 Adverse events of bevacizumab through IPC and IPC alone in patients with or without actionable mutation**

|  | Actionable Mutation (N=104) | | | Without Actionable Mutation (N=45) | | |
| --- | --- | --- | --- | --- | --- | --- |
| Adverse events | Bevacizumab  through IPC (n=21) | IPC  (n=83) | Total | Bevacizumab through IPC  (n=6) | IPC  (n=39) | Total |
| Pain | 2 (9.5) | 2 (2.4) | 4 (3.9) | 0 | 1 (2.6) | 1 (2.2) |
| Pleural infection | 0 | 2 (2.4) | 2 (1.9) | 0 | 0 | 0 |
| Fever | 0 | 1 (1.2) | 1 (1.0) | 1 (16.7) | 1 (2.6) | 2 (4.4) |
| Catheter blockage | 1 (4.8) | 1 (1.2) | 2 (1.9) | 0 | 1 (2.6) | 1 (2.2) |
| Tube dislodgement | 0 | 1 (1.2) | 1 (1.0) | 0 | 0 | 0 |
| Dyspnea | 0 | 0 | 0 | 0 | 1 (2.6) | 1 (2.2) |
| Catheter-associated  cellulitis | 0 | 2 (2.4) | 2 (1.9) | 0 | 1 (2.6) | 1 (2.2) |

IPC: indwelling pleural catheter; Actionable mutation: patients with sensitizing *EGFR* mutation or *ALK/ROS1* fusion.
